# Author’s Response to Peer Reviews of “Incidence of Postoperative Diabetes Mellitus After Roux-en-Y Reconstruction for Gastric Cancer: Retrospective Single-Center Cohort Study”

**DOI:** 10.2196/63859

**Published:** 2024-08-14

**Authors:** Tatsuki Onishi

**Affiliations:** 1 Data Science and AI Innovation Research Promotion Center, Shiga University, 1 Chome-1-1 BambaHikone, Shiga, 522-0069, Japan, 81 749 27 1030; 2 Department of Anesthesia, Juntendo University Shizuoka Hospital, Izunokuni, Shizuoka, Japan; 3 Department of Anesthesia, Kyowa Hospital, Kyoto, Japan

**Keywords:** diabetes mellitus, gastrectomy, gastric cancer surgery, glucose metabolism, postoperative diabetes onset, surgery outcomes

*This is the author’s response to peer-review reports for “Incidence of Postoperative Diabetes Mellitus After Roux-en-Y Reconstruction for Gastric Cancer: Retrospective Single-Center Cohort Study.”*

## Round 1 Review [1]

*This review is the result of a virtual, collaborative live review discussion organized and hosted by PREreview and JMIR Publications. The discussion was joined by 18 people: 2 facilitators, 4 members of the JMIR Publication team, and 12 live review participants. Konstantinos Georgiou, Maria Florencia Grande Ratti, and Naser Kamyari wished to be recognized for their participation in the live review discussion, even though they have not contributed to authoring the review below. We thank all participants who contributed to the discussion and made it possible for us to provide feedback on this preprint.*

### Summary

*This study [2] compares the results of Roux-en-Y (RY) reconstruction with other surgical techniques (OT) to determine the incidence of postoperative diabetes in patients with gastric cancer who had undergone total gastrectomy. The Tokyo Metropolitan Bokutoh Hospital cohort of 715 patients from 2005 to 2019 was examined. The study finds a statistically significant difference in the incidence of postoperative diabetes between the RY and OT groups, with RY associated with a greater incidence, through careful data preprocessing and statistical analysis. The study does admit many limitations, though, such as the absence of a control group that did not undergo a gastric bypass and the lack of assessment of the role that lifestyle factors and genetic predisposition play in the development of diabetes. The study also suggests more investigation into the possible effects of laparoscopic jejunal interposition reconstruction on gut flora and postoperative outcomes.*

*This retrospective, single-center study analyzed electronic medical records, which used hemoglobin A*_*1c*_ *(HbA*_*1c*_*) levels as a surrogate for the determination of diabetes status in patients. The study aimed to examine the incidence of new-onset diabetes in patients with gastric cancer who had undergone gastrectomy. Interestingly, the author presents the data via Kaplan-Meier curves, which describe a statistically significant difference, revealing that patients who had an RY reconstruction were more likely to develop new-onset diabetes than patients where surgical reconstruction was achieved via other techniques.*

*While the findings are interesting, it is essential to enhance the clarity of the study by providing additional information on the sampling methods, the determination of sample size, and a breakdown of the number of events in each group to enable an accurate understanding of study procedures and outcomes. Moreover, an analysis of patients at risk of diabetes before surgery would reduce potential confounding factors. This could be achieved by including a Cox proportional hazard regression to potentially provide more information on the impact of reconstruction methods for the risk of developing diabetes, while also accounting for other covariates. An explanation and breakdown of other reconstructive techniques (in the OT group) would improve the utility and external validity of this study. Additionally, the participants could have had other comorbidities that could affect the outcome. Therefore, a note on the inclusion criteria and exclusion criteria is necessary.*

*Below we list major and minor concerns that were discussed by participants of the live review, and where possible, we provide suggestions on how to address those issues.*

### List of Major Concerns and Feedback

*1. There was no rationale provided for the choice between RY and OT. Were any guidelines followed, or was this at the discretion of the attending physician?*

**Response:** The choices were made according to the preferences of the attending physician.

*2. Due to the complex nature of postoperative diabetes development, it is crucial to take any confounding variables into consideration and provide a full description of any adjustments made.*

**Response:** I included further detailed demographics (updated in Table 1), and to cope with confounding, I added a propensity score matching analysis.

*3. The author should consider including appropriate covariates in the study to assess if they have a confounding effect on the study’s result. For instance, is the author able to stratify the patients in terms of their risk of developing diabetes or include relevant information such as family history or concurrent metabolic syndrome?*

**Response:** I added further detailed information in Table 1, but unfortunately, as I have retired from the hospital, information other than what I have collected cannot be implemented.

*4. The author should explicitly state the study’s inclusion and exclusion criteria. Please consider giving more details on the comorbidities of the included participants. This could be summarized, or tools such as the Charlson Comorbidity Index could be used.*

**Response:** Like in response 3, I was not able to include this information. I am sorry for that.

*5. Sufficient details are not provided to allow the reproduction of the study; thus, we suggest you follow the STROBE (Strengthening the Reporting of Observational Studies in Epidemiology) guidelines for reporting. For example, there is content in the Methods section that should go in Results, such as the number of participants included and their baseline characteristics in Table 1. In the same way, information is missing in the Methods section, such as clear definitions of outcomes, statistical analysis, or sample size calculation.*

**Response:** I revised and reshaped the entire manuscript according to the STROBE guidelines.

*6. As the cumulative risk of bias for this type of study design is moderate to high, please identify all the variables used in the model. Clearly define all outcomes, exposures, predictors, potential confounders, and effect modifiers. Clarify the inclusion and exclusion criteria, together with follow-up time frames and intervals. As the patients underwent surgery between 2005 and 2019, may we assume that the shortest follow-up after surgery was 3 or 4 years?*

**Response:** I added descriptions for these.

*7. Also, describe any efforts to address potential sources of bias and explain how the study size was arrived at. Namely, the distribution of the age and sex of the participants is not clear, as there appears to be a bias toward male participants. Refer to the SAGER (Sex and Gender Equity in Research) guidelines for details on conducting a sex-based analysis and disaggregating data according to sex.*

**Response:** The distribution of the age and sex of the participants is clearly stated in Table 1, and I regenerated Table 1 for visuality. Thank you for mentioning the SAGER guidelines. I also added an analysis stratified by sex.

*8. Please report the regression model used to assess the associations between the explanatory variables and survival or time to event. How did the author handle learning effects, and the changing and evolving surgical or clinical protocols over the long time frame of this retrospective analysis? Discuss the generalizability of this modeling approach, as well as the direction and magnitude of any potential bias.*

**Response:** The development of surgical techniques during this period is not known. At least no development in surgical technique is known to be involved in the development of postoperative diabetes. No specific direction of bias was assumed, but propensity score matching was performed to address confounding bias.

*9. Please include explicit information regarding the competing outcome (ie, mortality events), and justify why no other clinical factors other than HbA_1c_ levels were considered.*

**Response:** Swimmer plots were added to visualize the occurrence of competing outcomes. The onset of diabetes was determined by either the name of the disease in the electronic health record or the HbA_1c_ level.

*10. How did the author confirm if the patients were free of diabetes at the time of surgery and before? It would be appropriate if the author provided the baseline (at the time of surgery or before) HbA_1c_ values of the study participants in Table 1.*

**Response:** Since HbA_1c_ is the default test item before surgery, we determined that the patient had diabetes if the HbA_1c_ test or the name of the disease in the electronic medical record mentioned diabetes. We did this by looking at the HbA_1c_ values when we extracted the cases, but we did not state it at the time, and I am sorry that I cannot look back and add it now that I have left the hospital in question.

*11. The discussion focused on a procedure that was not mentioned elsewhere or used in this study. Please clarify if this procedure is part of your recommendation for the clinical management of these patients in the future. Additionally, mention if future planned studies will address any stratification of patients for risk of new-onset diabetes mellitus prior to the surgery, or any analysis of gut microbiota before and after surgery.*

**Response:** The laparoscopic jejunal interposition reconstruction method is relatively new, but we believe that its implementation will increase in the future as no major problems have been identified in previous reports. There are plans to apply for access to the database at the prefectural level and conduct a similar but larger study to this one with a prefectural dataset. If this study is accepted, we intend to ask the database manager to provide the data with the results. Detailed data on gut microbiota, family history of diabetes, and diet are also expected to be included in the dataset that will be submitted for future use.

### List of Minor Concerns and Feedback

*12. Were any validation techniques used to verify the accuracy of the applied algorithms and analysis, such as code review, unit testing, or cross-validation?*

**Response:** As for code review, it has not been carried out, but the code is public on GitHub, so if something is obviously wrong, it will be pointed out. Also, because it is public, even if imperfections are found in the code in the future, it can be discussed in an open environment.

*13. It would be helpful to include a figure explaining the methodology, include more information about the proportion of different reconstructive techniques, and discuss results from other studies to attempt some comparisons for identifying what could have caused similarities or differences in this analysis. For instance, we do not know if the analysis of the groups was blinded.*

**Response:** Groups were not blinded; reviewed information will be included in Table 1.

*14. The Methods section lacks proper referencing of previous studies to justify the choice of reconstruction methods (RY vs OT) and the criteria used for defining the onset of diabetes. Referencing previous studies that have investigated similar surgical techniques or criteria for diabetes onset would provide the necessary context and justification for the methods used in the study. Additionally, citing relevant literature would enhance the credibility of the study by demonstrating that the research methodology is grounded in established practices and informed by prior research findings.*

**Response:** The choice of reconstruction method depends solely on the surgeon’s preference. For references, similar studies are cited in the *Introduction*—kindly refer to them.

*15. Clear visualization of censored data points on a Kaplan-Meier survival curve is essential for accurately interpreting the survival probabilities and understanding the impact of censoring on the analysis. Optionally, you can include CIs for the stratified number of participants.*

**Response:** A Swimmer plot was added.

*16. Due to the long time frame of the retrospective analysis and the possibilities of changes in protocol, the author should consider describing how learning effects were handled in the study.*

**Response:** No specific surgical procedures are known to be associated with the development of postoperative diabetes. In addition, the hospital is a training hospital, where surgeons rotate after an average of 2 years and are transferred to other teams (eg, stomach to colon) or to other hospitals, so learning changes are likely to be minimal.

*17. There should have been more information about the American Society of Anesthesiologists (ASA) score; this is a subjective score, so even if it was lifted from the electronic record, there ought to be a note pertaining to how many operators assigned the score and the degree of agreement between them.*

**Response:** Discrepancies in ASA scores are indeed a problem, and agreement is said to be around 40% according to the literature. In this paper, the distribution of ASA scores is presented as a reference instead of a comorbidity score. I understand that this should be the Charlson Comorbidity Index or a more diabetes-specific risk and comorbidity assessment, but having left the institution, this is my best-available measure.

*18. It is unclear how the missing values were handled. Were they imputed based on a model? What was the definition of an outlier here: greater than 2.5 SDs? What data types are being referred to here? And what inconsistencies needed to be corrected?*

**Response:** In the statistical statement, the treatment of outliers was mentioned, but this statement was deleted because no cases were actually excluded as outliers.

*19. What happened to the study participants after 2008 in the OT group (Figure 1)? Why is there a straight line?*

**Response:** The straight line is due to the absence of further diabetes onset in the OT group. A Swimmer plot has been added to make this easier to understand.

*20. Please provide more detailed information on what the code does in this study and how it could be used elsewhere.*

**Response:** The entire code has been uploaded to GitHub and is public, so anyone can verify it.

*21. In the Abstract, the study setting has been indicated as “Electrical medical records.” It should be “Electronic medical records*.”

**Response:** Thank you for pointing that out.

### Concluding Remarks

*We thank the author of the preprint for posting their work openly for feedback. We also thank all participants of the live review call for their time and for engaging in the lively discussion that generated this review.*

**Response:** I changed the colors of lines for visibility. Red (orange) for RY and blue (light blue) for OT.

There is a linear section due to the absence of events, but if this is an obstacle to understanding, it could be replaced by one of shorter duration. The hazard ratios have been calculated for all data, so the various statistics would not change, just that the Kaplan-Meier curve is easier to understand visually. In Figure 1, both intervals are presented for reference.

## Abbreviations

ASA: American Society of Anesthesiologists
HbA_1c_: hemoglobin A_1c_
OT: other surgical techniques
RY: Roux-en-Y
SAGER: Sex and Gender Equity in Research
STROBE: Strengthening the Reporting of Observational Studies in Epidemiology
